# Cardiac Complications in Trisomy 21 Patients in a Secondary Hospital: A Descriptive Study

**DOI:** 10.7759/cureus.58058

**Published:** 2024-04-11

**Authors:** Mohammed AlDar, Ali Almaa, Hussain Alturki, Hawra S Alkhalifah, Walaa H AlSaeed, Ahmed S Alkhalifah

**Affiliations:** 1 Pediatrics and Neonatology, Qatif Central Hospital, Qatif, SAU; 2 Pediatrics, Qatif Central Hospital, Qatif, SAU; 3 Public Health, General Directorate of Health Affairs, Dammam, SAU; 4 Pediatric Critical Care, Qatif Central Hospital, Qatif, SAU

**Keywords:** down syndrome (ds), kingdom of saudi arabia (ksa), cardiac echo, pediatric congenital heart disease, pediatric

## Abstract

This retrospective study addresses the intersection of Down syndrome (DS) and congenital heart defects (CHD), including the prevalence and characteristics of CHD in trisomy 21 patients at a secondary hospital in the Eastern Province of Saudi Arabia. The study investigated the electronic medical records retrieved from the Qatif Central Hospital database, specifically targeting individuals diagnosed with DS (identified by the International Classification of Diseases, Tenth Revision (ICD-10) code Q90.9) between March 2012 and March 2022. The primary aim was to detect cardiac anomalies diagnosed via echocardiography performed at the hospital, along with subsequent follow-up assessments and documented patient outcomes.

Among the 161 patients reviewed, the study revealed a significant prevalence of diagnosed heart defects through echocardiograms, constituting approximately 72.7%. Notably, patent ductus arteriosus was the most common condition, found in 29.81% of cases, followed by atrial septal defect (27.95%) and atrioventricular septal defect (17.39%).

Among the study participants, 22.98% required surgical intervention. Unfortunately, mortality impacted 32.3% of individuals, while the majority (60.87%) remained alive. In addition, a small percentage (6.83%) discontinued follow-up within our center.

This study contributes significant data on cardiac anomalies in DS patients in Saudi Arabia, highlighting a high prevalence of CHD with specific patterns of anomalies. The need for early diagnosis, timely surgical intervention, and ongoing management is evident. These findings provide a foundation for improving clinical practices and shaping public health policies tailored to the needs of this population in Saudi Arabia and similar regions.

## Introduction

Down syndrome (DS) is the most common chromosomal disorder among live births; it is also the most common chromosomal disorder causing intellectual disability [1]. It is an aneuploidy caused by a trisomy of part of or all of chromosome 21 [2]. Trisomy 21 occurs either by non-disjunction, in which there is a supernumerary free copy of chromosome 21, translocation, when an additional chromosome 21 is translocated in another chromosome, and finally, mosaicism or partial trisomy 21, both of which are very rare and associated with fewer phenotypical manifestations [2,3].

DS affects approximately one in 800 live births worldwide and around one in 500 live births in the United States [1]. In Saudi Arabia, a nine-year study conducted from 1982 to 1991 found the incidence to be one in 554 live births, with all but one having non-disjunction in chromosomal analysis, while the last patient had translocation [4].

The phenotypic manifestation of DS includes a wide array of symptoms affecting multiple body systems [1,3]. These include craniofacial anomalies, which give the typical features of DS infants, such as epicanthal folds, up-slanted palpebral fissures, small mouths, and large tongues. Other manifestations include neurological, developmental, hematological, respiratory, endocrinological, autoimmune, and cardiovascular [3,5].

A congenital heart defect (CHD) is a major cause of morbidity and mortality among individuals with DS [6]. CHD affects approximately 50% of patients with DS, which is caused by the abnormal development of the heart during intrauterine life. The most common CHD affecting patients with DS is atrioventricular septal defect (AVSD), followed by atrial septal defect (ASD) and ventricular septal defect (VSD) [7]. If CHD is not managed properly, it could lead to lifelong complications such as pulmonary hypertension, which is found in up to one out of five patients with DS, emphasizing the need for early diagnosis and management of CHD [8]. Innovation and improvement in the detection and management of CHD in the past decades have increased the life expectancy of DS patients from 30 years to 60 years [1].

This single-center study aims to illustrate the incidence and patterns of CHD in the eastern province and to compare it to previously published national and international data.

## Materials and methods

This retrospective study reviewed the electronic medical records (EMRs) of DS patients diagnosed with heart defects via echocardiography at our center. The EMRs were reviewed to look at heart defects through cardiology reports, any follow-up studies, and the documented outcomes for the patients. Qatif Health Network Institutional Review Board approved the study (QCH-SREC0 37/2022).

Study population

The Qatif Central Hospital (QCH) database was accessed to retrieve a list of patients coded with DS according to the International Classification of Diseases, Tenth Revision (ICD-10) code Q90.9 from March 2012 to March 2022. A total of 292 patients fulfilling the criteria of having DS as a diagnosis and undergoing echocardiography at least once in their lifetime were reviewed; of these, 161 patients were included in the final analysis. The remaining patients were excluded due to the lack of comprehensive data in their EMRs.

For each patient included in the study, demographic data and relevant clinical information were extracted from their EMRs. This information included age at diagnosis, specific cardiac lesions identified, any associated comorbidities, undertaken cardiac interventions, and the outcomes for these patients. A specially designed datasheet was utilized for logging this data to ensure systematic collection and analysis.

Data analysis

Data analysis was performed using Python 3.7 (Python Software Foundation, Wilmington, Delaware, USA). The initial step involved reviewing and cleaning the dataset to ensure data accuracy and consistency. Following data preparation, descriptive statistics were generated to provide an overview of the study population using median values and interquartile ranges (IQR). Categorical variables were presented as counts and percentages.

To investigate the prevalence of cardiac abnormalities within our study cohort, we created bar charts illustrating the distribution of various cardiac defects, the surgeries undertaken, and the prevalence of comorbidities. This visual representation aimed to provide clear insights into the patterns of cardiac issues and their management in patients with DS.

Finally, for the purpose of examining the relationships between the types of cardiac defects, the interventions performed, comorbidities, and mortality rates, we employed Fisher's exact test. This statistical test was chosen for its efficacy in analyzing categorical data, especially useful in studies with smaller sample sizes or when the data distribution does not meet the assumptions required for the chi-square test.

## Results

A total of 292 patients labeled as having DS were reviewed. One hundred sixty-one patients were included in the analysis. The rest were excluded from the analysis due to missing data (no records of echo results were found).

Table 1 shows the descriptive analysis of the study population. The table includes information on birth height, gestational age, age at first cardiac intervention, family history of congenital heart disease, cardiac follow-up per year, and outcome.

**Table 1:** Descriptive analysis of the study’s population IQR: interquartile range, DS: Down syndrome

Patient charactartistc	Results
Birth height in cm; median (IQR)	49.0 (46.0-50.0)
Birth weight in kg; median (IQR)	2.7 (2.4-3.1)
Gestational age; median (IQR)	38.0 (37.0-40.0)
Age of first cardiac intervention; median (IQR)	0.0 (0.0-1.0)
Number of cardiac interventions; median (IQR)	1.0 (1.0-1.0)
Age at diagnosis days; median (IQR)	5.0 (1.0-140.0)
Age at diagnosis months; median (IQR)	0.17 (0.03-4.67)
Gender: male; n (%)	89.0 (55.28%)
Nationality: Saudi; n (%)	148.0 (91.93%)
Age group: pediatrics; n (%)	136.0 (84.47%)
Family history of DS; n (%)	
Unknown	115.0 (71.43%)
No	37.0 (22.98%)
Yes	9.0 (5.59%)
Family history of congenital heart disease; n (%)	
Unknown	123.0 (76.40%)
No	35.0 (21.74%)
Yes	3.0 (1.86%)
Consanguinity; n (%)	
Unknown	117.0 (72.67%)
No	38.0 (23.60%)
First cousins	3.0 (1.86%)
Second cousins	3.0 (1.86%)
Required surgical intervention; n (%)	
No	124.0 (77.02%)
Yes	37.0 (22.98%)
Cardiac evaluations per year; n (%)	
Once	50.0 (61.73%)
Twice	19.0 (23.46%)
3 times	6.0 (7.41%)
4 times	5.0 (6.17%)
≥5 times	1.0 (1.23%)
Outcome; n (%)	
Alive	98.0 (60.87%)
Dead	52.0 (32.30%)
Lost follow-up	11.0 (6.83%)

**Figure 1:**
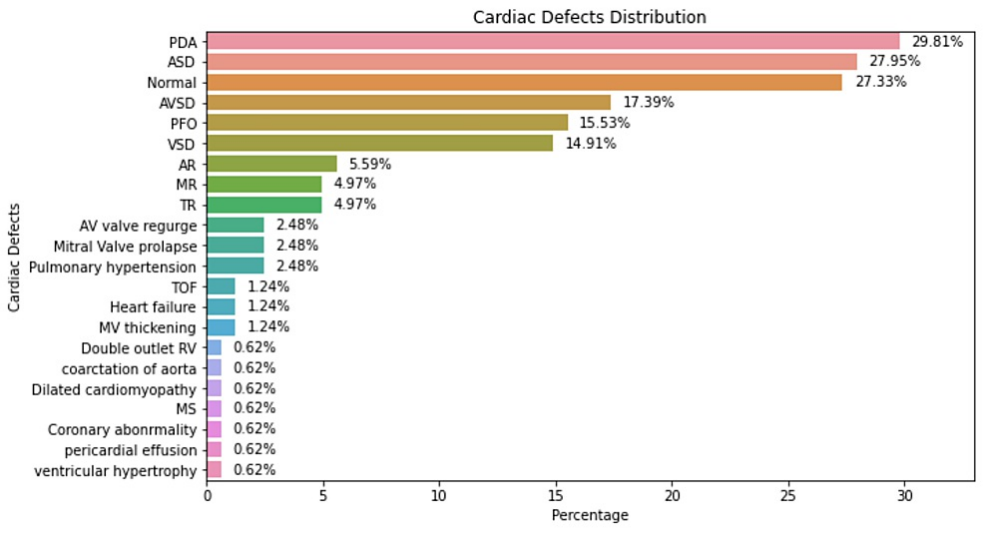
The most common cardiac defect among DS patients enrolled in the study DS: Down syndrome, PDA: patent ductus arteriosus, ASD: atrial septal defect, AVSD: atrioventricular septal defect, PFO: patent foramen ovale, VSD: ventricular septal defect, AR: aortic regurgitation, MR: mitral regurgitation, TR: tricuspid regurgitation, AV: aortic valve, TOF: tetralogy of Fallot, MV: mitral valve, RV: right ventricle, MS: mitral stenosis

In this study cohort of DS patients, patent ductus arteriosus (PDA) emerged as the most prevalent cardiac anomaly, accounting for 29.81% of cases. In contrast, ventricular hypertrophy was the least common, with a mere 0.62%. The study also identified a range of other cardiac conditions, including ASD, VSD, patent foramen ovale, aortic regurgitation, mitral regurgitation, pulmonary hypertension, mitral valve prolapse, tetralogy of Fallot, heart failure, right ventricle outflow tract obstruction, coarctation of the aorta, dilated cardiomyopathy, coronary artery disease, and pericardial effusion. Figure 1 illustrates the frequency of various heart defects in the study's DS participants.

**Figure 2:**
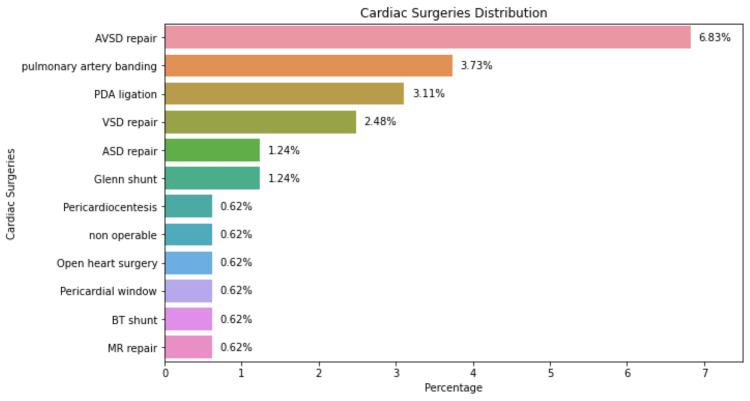
Cardiac intervention distribution AVSD: atrioventricular septal defect, PDA: patent ductus arteriosus, VSD: ventricular septal defect, ASD: atrial septal defect, BT shunt: Blalock-Taussig shunt, MR: mitral regurgitation

Regarding surgical interventions in this study cohort, the repair of AVSD was the most frequently performed cardiac surgery, constituting 6.83% of all procedures. This was closely followed by pulmonary artery banding at 3.73% and PDA ligation at 3.11%. On the lower end of the spectrum, surgeries such as MR repair, Blalock-Taussig shunt, creation of a pericardial window, open heart surgery, Glenn shunt, and placement of a stent each accounted for 0.62% of the procedures. Figure 2 presents an overview of the distribution of various cardiac surgeries conducted on the DS patients in the study.

**Figure 3:**
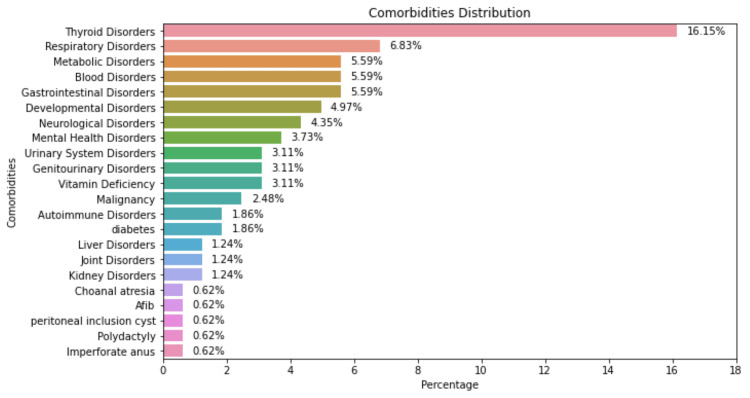
The distribution of comorbidities found among the study population Afib: atrial fibrillation

Figure 3 shows the distribution of comorbidities among the DS patients included in the study. The most common comorbid conditions were thyroid disorders, with a percentage of 16.15%, while imperforate anus had the lowest percentage of 0.62%. Other comorbidities included respiratory, developmental, gastrointestinal, blood, mental, neurological, and urinary system disorders, vitamin deficiency, malignancy, liver disorders, diabetes, autoimmune disorders, renal disorders, cholangitis, and kidney inclusion cysts.

This descriptive study provides valuable information on the prevalence and distribution of cardiac defects among DS patients in QCH. The findings can be used to identify this population's most common cardiac defects and comorbidities and develop targeted interventions to improve health outcomes.

## Discussion

Overview and contextualization of findings

This study at QCH investigated the cardiac complications in DS patients, revealing a high prevalence of CHD in this population. The most commonly observed cardiac anomalies were PDA, ASD, and AVSD, with a significant portion requiring surgical intervention. These findings not only corroborate previous research but also offer new insights into the cardiac manifestations of DS in the Saudi Arabian context.

Comparison with global and regional data

The prevalence of CHD in our study (72.7%) was almost consistent with the previous reports that CHD is a common comorbidity in DS. For example, Freeman et al., in 1998, reported a similar prevalence, emphasizing the ubiquity of CHD among DS patients globally [9]. However, the distribution of CHD types in our study was not compatible with the pattern reported in some of the previous international and national studies (AVSD is the most common type of cardiac defect in DS), which may be attributed to the variation in the genetic or environmental factors as it was compatible with some of the previous studies in Saudi Arabia (PDA is the most common cardiac defect in DS) [4,8,10-14]. Recognizing these regional discrepancies is essential for developing tailored healthcare strategies, including specific screening and management protocols, to effectively address the varying needs of DS patients nationwide.

Surgical interventions and outcomes

The need for surgical intervention in this study (22.98%) reflects the critical nature of managing CHD in DS patients. The literature underscores the evolution of surgical techniques that have significantly improved outcomes [6,13,15,16]. However, our study's mortality rate (32.3%) remains a concern and may reflect broader issues such as parents' awareness, access to care, or early diagnosis delays. This aspect warrants further exploration, as emphasized by Marder et al., who discuss the importance of early and effective intervention in this patient population [6].

Implications for clinical practice and public health

The high prevalence of CHD in DS patients in our study underscores the need for routine cardiac screening and early intervention strategies. This approach aligns with Marder et al., who advocate for early echocardiographic screening for all DS patients [6]. The variation in CHD types observed in our study compared to global data suggests that region-specific screening protocols and management strategies may be beneficial.

Future research directions

While our study provides valuable insights, it is imperative to recognize its limitations, including its retrospective nature and focus on a single hospital. Future research should aim for multicenter studies with larger sample sizes to validate our findings and explore the long-term outcomes of DS patients with CHD. Additionally, research into the genetic and environmental factors contributing to the regional differences in CHD prevalence and types could yield important insights.

Limitation

This study’s main limitation was the study design; being a retrospective study means that there are missing data and patients, which limits the preciseness of the results. Additionally, the lack of a fetomaternal specialty at QCH may restrict antenatal diagnostics, potentially excluding key patient data. This may affect the results of this study. More prospective multicenter studies are needed to be conducted to assess the prevalence, distribution, and outcome of CHD in DS in Saudi Arabia.

## Conclusions

This study contributes to the data on cardiac anomalies in DS patients in Saudi Arabia, highlighting a high prevalence of CHD with specific patterns of anomalies. The need for early diagnosis, timely surgical intervention, and ongoing management is evident. To better understand the full spectrum of DS and its impacts, we advocate for establishing national registries in Saudi Arabia. These registries would provide a holistic overview of all DS-related conditions, enhancing our ability to monitor, research, and respond to this population's unique healthcare needs. These findings provide a foundation for improving clinical practices and shaping public health policies tailored to the needs of this population in Saudi Arabia and similar regions.
